# Primary cilia in the mature brain: emerging roles in Alzheimer’s disease pathogenesis

**DOI:** 10.3389/fcell.2025.1650884

**Published:** 2025-09-24

**Authors:** Alexis Shiying Huang, Amy Sze Man Li, Catherine Hong Huan Hor

**Affiliations:** ^1^ Department of Chemistry, Hong Kong Baptist University, Hong Kong, Hong Kong SAR, China; ^2^ Department of Neurobiology, Care Sciences and Society, Karolinska Institute, Huddinge, Sweden; ^3^ Consun Chinese Medicines Research Centre for Renal Diseases, School of Chinese Medicine, Hong Kong Baptist University, Hong Kong, Hong Kong SAR, China

**Keywords:** primary cilia, Alzheimer’s disease, pathogenesis, G protein-coupled receptors, Aβ plaques

## Abstract

Primary cilia are microtubule-based structures that resemble antennae and function as sensory organelles. Dysfunction of primary cilia has been linked to various age-related conditions. Alzheimer’s disease, which affects more than 38.5 million individuals worldwide, is a prominent neurodegenerative disorder, with aging being its most significant risk factor. In this review, we provide an overview of current findings on the role of primary cilia in the mature brain and the mechanisms by which alteration of primary cilia may influence the progression of Alzheimer’s disease. Growing evidence reveals that primary cilia in the mature brain play dynamic roles in cell type, region, and age-dependent manners. In Alzheimer’s disease, anomalies in primary cilia functions and morphology are closely associated with key pathologies. However, the exact mechanisms remain unclear. Future studies on neuronal and glial cilia dynamics during aging and neurodegeneration are essential to explore their potential as therapeutic targets.

## Introduction

Alzheimer’s disease (AD) is a neurodegenerative disorder characterized by the presence of amyloid-β (Aβ) plaques and tau neurofibrillary tangles, resulting in progressive cognitive impairment, with aging being the most significant risk factor (Knopman et al., 2021). Beyond Aβ and tau pathology, AD progression involves disruptions in cellular homeostasis, notably affecting endosomal-lysosomal clearance (Nixon, 2017). Recent research has implicated primary cilia dysfunction in the pathogenesis of AD (Ma et al., 2022), suggesting that these organelles play a critical role in aging and age-related brain disorders.

The primary cilium is a microtubule-based, non-motile organelle that extends from the surface of most mammalian cells (Wheatley et al., 1996). The ciliary membrane extends continuously from the plasma membrane but is notably enriched with ion channels, G-protein-coupled receptors (GPCRs), and essential components of signaling pathways like Sonic Hedgehog (Shh) and Wnt (Pala et al., 2017). This composition allows the primary cilium to serve as a non-synaptic sensory and signaling organelle. The primary cilium is also a dynamic structure, with its assembly and disassembly regulated during the cell cycle and in response to developmental cues (Plotnikova et al., 2009).

Primary cilia play essential roles in brain development and neurogenesis (Hasenpusch-Theil and Theil, 2021). Notably, primary cilia function as essential signaling hubs for pathways such as Shh, which is crucial for neural tube patterning and the expansion of neural progenitor populations (Zaidi et al., 2022). In the mature brain, primary cilia are typically located on the soma or dendrites of neurons (Wu et al., 2024), where they serve as sensory organelles that integrate local environmental cues, thereby influencing neurotransmission and synaptic plasticity. For example, primary cilia are enriched with neuropeptide receptors such as somatostatin receptor 3 (SSTR3), which modulate excitatory synaptic input onto neocortical pyramidal neurons (Tereshko et al., 2021). Additionally, proper cilia assembly is necessary for the formation of glutamatergic synapses with entorhinal cortical projections, as demonstrated by Kumamoto et al. in newborn dentate granule cells of 5-week-old female C57 mice (Kumamoto et al., 2012).

Intriguingly, recent studies begin shedding lights on the key mechanisms that connect primary cilia dysfunction to AD progression. This review integrates studies using cells from the cortex and hippocampus of rodents and humans, alongside rodent models, to elucidate the primary cilia’s roles in the central nervous system. We discuss the heterogeneity in morphology in the mature brain, with an emphasis on variations across cell types, brain regions, and age-related changes. We also review current findings on the functions of neuronal ciliary-localized GPCRs. Furthermore, we highlight the relevance of primary cilia to AD pathology and identify key gaps in knowledge that warrant further investigation.

## The structure of the primary cilium

The primary cilium is a complex organelle composed of the basal body, axoneme, and transition zone.

The basal body is evolutionarily conserved, derived from the mother centriole, and is crucial for the initial assembly of the cilium. It consists of a barrel-shaped structure made of nine triplet microtubules, along with subdistal appendages and nine strut-like distal appendages (also called transition fibers). These distal appendages anchor the basal body to the membrane at the cilium’s base (Reiter and Leroux, 2017).

The axoneme, surrounded by a membrane that is continuous with the plasma membrane, consists of nine circumferentially arranged microtubule doublets that extend from the basal body into the extracellular space. This structure can vary along the length of the cilium, with the number of microtubule doublets and the diameter decreasing towards the tip (Sun et al., 2019; Wu et al., 2024).

Between the basal body and the axoneme, a region called transition zone serves as gatekeeper to control proteins in or out of the cilium (Lechtreck, 2015). The transportation of proteins to the primary cilium membrane is a tightly regulated process. The transition zone contains distinct Y-shaped structures that link the ciliary membrane to the axoneme. These structures are believed to establish or maintain a diffusion barrier, regulating the movement of membrane-associated soluble proteins (Awata et al., 2014). The selective function of the transition zone relies on multiple complexes (Park and Leroux, 2022). Apart from these complexes, intraflagellar transport (IFT) trains are responsible for the bidirectional transport of proteins and other molecules along the axonemal microtubules of the cilium (Hesketh et al., 2022). This system consists of two main complexes: IFT-A (e.g., IFT140) and IFT-B (e.g., IFT88, IFT20), alongside motor proteins kinesin-2 and dynein-2, facilitate the active transport of membrane proteins, such as receptors and ion channels, into and out of the cilium (Klena and Pigino, 2022; van den Hoek et al., 2022). IFT trains bind to traffic cargos either directly or via associating factors. IFT-A interacts with tubby family protein 3 (TULP3) to promote GPCRs trafficking into the cilium (Mukhopadhyay et al., 2010; Badgandi et al., 2017). The BBSome, a protein complex composed of eight Bardet-Biedl syndrome (BBS) proteins and directly interacts with IFT subcomplexes, is required for retrograde trafficking of GPCRs in and out of primary cilia (Berbari et al., 2008; Wingfield et al., 2018; Ye et al., 2018). Together, these components underscore the intricate interplay of transport, sorting, and stabilization that defines the primary cilium as a specialized signaling hub (Wei et al., 2012).

## Primary cilia in the mature brain

In the human brain, primary cilia occupy 0.03% of cortical volume (Wu et al., 2024). For a long time, it was believed that primary cilia were present in neurons and astrocytes in the brains of adult humans and rodents (Bishop et al., 2007; Yoshimura et al., 2011; Wu et al., 2024), but were absent from microglia and oligodendrocytes (Sipos et al., 2018). This understanding has been recently updated by findings that demonstrate the presence of primary cilia on microglia (Liao et al., 2023; Yeo et al., 2023). Primary cilia can also be found in choroid plexus cells in the adult mouse brain (Bishop et al., 2007).

Primary cilia in the brain can be identified using molecular markers such as adenylate cyclase 3 (AC3), ADP-ribosylation factor-like protein 13B (ARL13B), and certain GPCRs, all of which are enriched within the ciliary membrane. However, their expression exhibits cell-type-specific variability. AC3 is widely used to label neuronal primary cilia, although it also labels a subset of astrocytic cilia in the human, rat, and mouse cerebral cortex (Yoshimura et al., 2011; Sipos et al., 2018). In contrast, Arl13b is more commonly used for astrocytic cilia but has also been detected in a limited population of neuronal cilia (Sipos et al., 2018; Dupuy et al., 2023). Quantitative analyses in adult C57 mice show that 78% of neurons exhibit AC3-positive primary cilia, while 47% are Arl13b-positive; conversely, 88% of astrocytes are Arl13b-positive, and nearly half express AC3-positive cilia (Kasahara et al., 2014). Regional differences have also been reported. In the mouse brain, for example, only 38% of astrocytes in the corpus callosum are Arl13b-positive, despite most astrocytes elsewhere bearing a single Arl13b-labeled primary cilium (Wang et al., 2024). Several GPCRs, including SSTR3, melanin-concentrating hormone receptor 1 (MCHR1), and 5-hydroxytryptamine receptor 6 (5-HT_6_ receptor), also serve as markers for neuronal primary cilia and have been used to investigate cilia-dependent signaling pathways (Domire and Mykytyn, 2009). For instance, an *in vitro* study using primary hippocampal cultures has demonstrated the presence of primary cilia on both neurons and astrocytes from newborn mice after 7 days of serum-free culture (Berbari et al., 2007). In this study, cilia were visualized using two common markers: AC3 and Sstr3. Notably, the choice of marker had a significant influence on cilia detection. In neurons, AC3 labeled nearly three times more cilia than Sstr3. Furthermore, while approximately 50% of astrocytes exhibited AC3-positive primary cilia, Sstr3 was completely absent from these cells (Berbari et al., 2007). These findings highlight not only the utility of primary cell culture in studying cilia and the importance of selecting appropriate markers, as detection can vary substantially across cell types and between different markers within the same cell population.

### Primary cilia in distinct neuronal subtypes and brain regions

Primary cilia in the brain exhibit notable morphological variations, including differences in length, spatial distribution, and microtubule architecture.

Neuronal primary cilia, unlike the typical 9 + 0 microtubule arrangement, display highly diverse microtubule architectures, with variable numbers of microtubule doublets along their length (Sheu et al., 2022; Wu et al., 2024). Furthermore, proteins with diverse lifespans coexist within the basal body and axoneme of neuronal primary cilia, suggesting dynamic turnover (Arrojo et al., 2019). In neurons, primary cilia exhibit diverse characteristics across brain regions and subtypes. In the cerebral cortex, primary cilia of upper-layer neurons are significantly longer than those in lower layers. Quantitative analysis reveals that interneuron primary cilia have an average length of 5.8 ± 0.2 µm, while projection neuron cilia measure 7.4 ± 0.6 µm on average. Most cortical neuronal cilia are positioned closer to dendrites than axons, suggesting a functional orientation, and up to 56% possess a ciliary pocket at their base, indicative of active vesicle transport and docking at the ciliary base (Wu et al., 2024). In the hippocampus of adult (3–8 months old) male mice, AC3-labeled primary cilia are abundant in the pyramidal cell layers of the CA1 and CA3 regions, as well as in the granular cell layer of the dentate gyrus (DG) (Wang et al., 2011). Within the CA1-CA3 molecular layer, neuronal primary cilia are radially oriented, with those in CA1 being the longest, averaging 8.9 µm, and typically originating from the soma (Vien et al., 2023). Additionally, 5-HT_6_ receptor-rich primary cilia of CA1 pyramidal neurons form synaptic connections with brainstem serotonergic axons, with approximately 35% of neuronal primary cilia in close apposition to these axons, accounting for about 50% of axon-ciliary synapses (Sheu et al., 2022). This suggests a specialized role of primary cilia in serotonergic signaling.

Primary cilia on astrocytes also display significant morphological plasticity. Astrocyte primary cilia showed a regional difference in length, with the longest found in the CA1 region of the hippocampus, averaging 3.8 ± 0.07 µm (Wang et al., 2024). Ciliary morphology in astrocytes is also sensitive to their physiological state. In C3-positive reactive astrocytes, primary cilia become significantly longer, even though the rates of cilia formation remain unchanged (Muhamad et al., 2024).

Recent studies have investigated how ciliary length and ciliation rates vary with the age of mice. In the neocortex of CD1 strain mice, the length of AC3-labeled primary cilia varies by cortical layer and reaches its full extent after 3 months, with no significant gender differences observed (Arellano et al., 2012). A study using sv129/C57/BL6 female mice reported that ciliary length and ciliation rates of Sstr3-labeled primary cilia in the dentate granule cell layer remain stable from 6 to 24 months of age (Chakravarthy et al., 2012). However, a study shows clear age-related changes in the hippocampus. In C57 BL/6J mice, primary ciliary lengths in the DG, CA1, and CA3 regions increased significantly from 6 to 15 months of age (CA1 and CA3: AC3/Mchr1 double-labeled, DG: AC3-labeled) (Kobayashi et al., 2022). Similarly, in aged male F344 x BN rats, AC3-labeled primary cilia in the CA1 and CA3 hippocampal regions are longer compared to young rats, while no changes in the ciliation rates and ciliary length were observed in the neocortex and DG (Guadiana et al., 2016).

### Neuronal G protein-coupled receptors on the ciliary membrane and their functional impact

**FIGURE 1:**
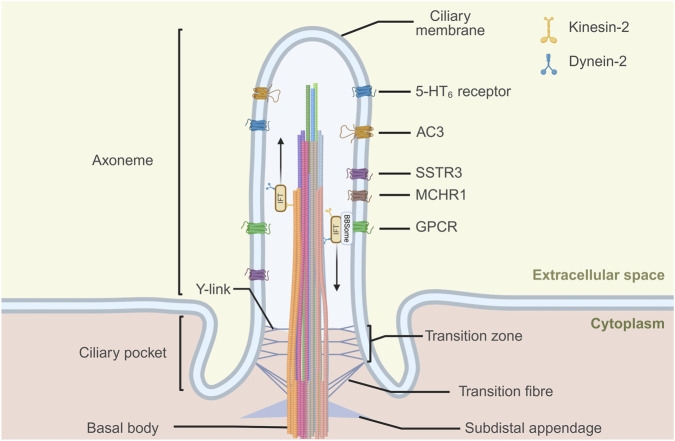
G protein-coupled receptors (GPCRs) express at neuronal primary cilia. The neuronal primary cilium consists of the basal body, axoneme, and transition zone. Its microtubule architecture is highly diverse, with variable numbers of doublets along the ciliary shaft. Protein entry and exit are regulated by the transition zone and transition fibers, together with the intraflagellar transport (IFT) machinery, which mediates kinesin-2–dependent anterograde and dynein-2–dependent retrograde transport. The BBSome complex acts as a cargo adaptor for retrograde trafficking of transmembrane proteins, including GPCRs. Neuronal primary cilia can be visualized with markers such as adenylate cyclase 3 (AC3) and several ciliary GPCRs, including somatostatin receptor 3 (SSTR3), melanin-concentrating hormone receptor 1 (MCHR1), and 5-hydroxytryptamine receptor 6 (5-HT_6_ receptor). Created in BioRender.

GPCRs localized to the ciliary membrane of neurons play critical roles in signaling. Notable GPCRs identified on primary cilia include MCHR1, SSTR3, and 5-HT_6_ receptor, which regulate processes such as memory, synaptic plasticity, and neuronal excitability (Domire and Mykytyn, 2009) (Figure 1).

MCHR1 is extensively found in the brains of mice and rats, showing a very similar distribution (Diniz et al., 2020). In the mouse brain, Mchr1 localizes to neuronal cilia across various regions (Green et al., 2012; Diniz et al., 2020). In the cerebral cortex, Mchr1 exhibits a layer-specific distribution that spans layers 2 to 6 (Diniz et al., 2020). Its presence is particularly notable in the hippocampus, where Mchr1-positive primary cilia are abundant in the stratum pyramidale of the CA regions, showing the greatest density in CA1, a reduced density in CA2, and minimal presence in CA3 (Diniz et al., 2020). This pattern is mirrored in rats, where Mchr1 is prominent in the neuronal cilia of CA1, and CA3 regions, but not in the DG. Notably, nearly all cilia in the CA1 region that express AC3 also co-express Mchr1. The correlation is striking in the rat CA1 region, where over 87% of AC3-labeled neuronal cilia are also Mchr1-positive, whereas only about 2% of AC3-labeled neuronal cilia in the DG co-express Mchr1. The length of Mchr1-bearing cilia also varies by region, being significantly longer in CA1 than in CA3 and the DG (Kobayashi et al., 2021). The trafficking of MCHR1 to the primary cilium is a tightly regulated process. Jouberin, encoded by the Abelson-helper integration site 1 (*AHI1*), localized at the ciliary transition zone, is essential for this process in neurons. In *Ahi1* ^−/−^ mice, primary cilia are fewer but longer in the hippocampus, with reduced Mchr1 ciliary localization, though total Mchr1 levels and plasma membrane trafficking remain unaffected (Hsiao et al., 2021). The protein tubby is also involved for moving Mchr1 to the primary cilia of hippocampal neurons (Sun et al., 2012). MCHR1 signaling is involved in ciliary morphology alteration. In rat brain slices, treatment with melanin-concentrating hormone (MCH) induces Gi/o- and Akt-dependent shortening of cilia in the CA1 region, but not cilia in the CA3. Fasting for 48 h in C57 mice did not change Mchr1 mRNA expression level, but reduced cilia lengths in the CA1 region (Kobayashi et al., 2021). In the mouse hippocampus (CA1 and CA2), within the neuronal primary cilia, Mchr1 colocalizes and interacts with Sstr3 (Green et al., 2012), suggesting functional cross-talk between signaling pathways.

Among the five somatostatin receptor subtypes, SSTR3 is unique for its selective localization to neuronal cilia (Schulz et al., 2000; Einstein et al., 2010; Wang et al., 2025). Tubby is reported to be essential for Sstr3 trafficking to primary cilia of neurons in the hippocampus and some cortical regions (Sun et al., 2012). In adult Wistar rat brains, Sstr3-marked cilia are present in several regions, including the cerebral cortex, the pyramidal layer of the hippocampal CA regions, and the granule cell layer of the DG (Händel et al., 1999). While an earlier study reported that *Sstr3*-knockout does not alter the number or overall structure of primary cilia in the CA1 region of mice (Einstein et al., 2010), recent work by Wang et al. demonstrated that Sstr3 specifically regulates ciliary length. Overexpression of Sstr3 in mouse primary hippocampal neurons elongated primary cilia in a dose-dependent manner, whereas knockdown markedly shortened them (Wang et al., 2025). Primary ciliary Sstr3 is essential for cognitive and synaptic function. Einstein et al. reported that *Sstr3*-knockout mice exhibit significantly reduced basal cyclic adenosine monophosphate (cAMP) levels in the hippocampus, impaired object recognition memory with a longer retention interval (1 h), and disrupted forskolin-induced long-term potentiation (LTP) compared to wild-type mice (Einstein et al., 2010). Wang et al. provided similar evidence, showing that *Sstr3* deletion impairs spatial memory in the Morris water maze, decreases LTP, reduces both the amplitude and frequency of miniature excitatory post-synaptic currents (mEPSCs), and leads to loss of dendritic spines, affecting both the number and volume of mushroom spines in the CA1 region. Sstr3 also regulates axon initial segment (AIS) structure and plasticity: knockout shortens axon initial segment length and abolishes positional plasticity, while re-expression restores axon initial segment morphology. Similar axon initial segment length changes were observed upon manipulation of other ciliary proteins, including 5-HT_6_ receptor and Ift88. Kyoto Encyclopedia of Genes and Genomes (KEGG) analysis of wild-type mice and *Sstr3*-knockout mice revealed that *Sstr3* deletion significantly affects multiple regulatory pathways, including endocytosis, PI3K-Akt signalling, and cAMP signalling. These disruptions reduce Akt-dependent cyclic AMP-response element binding protein (CREB)-mediated transcription at the axon initial segment, specifically downregulating ankyrin G, the master organiser of the axon initial segment. Hippocampal neurons from *Sstr3*-knockout mice displayed diminished cilia-related calcium dynamics and took longer to reach peak response compared to wild-type neurons. Since calcium signalling reflects ciliary activity, these results suggest that Sstr3 is essential for ciliary function (Wang et al., 2025). Sstr3 has also been observed to colocalize with the p75 neurotrophin receptor (p75^NTR^) in the primary cilia of cells in the cortex and hippocampus, with peak abundance in the DG (Chakravarthy et al., 2010). However, the functional significance of their interaction remains to be explored.

The 5-HT_6_ receptor is primarily found on the ciliary membranes of neurons in the brain (Hamon et al., 1999; Brailov et al., 2000; Sheu et al., 2022). Studies on 5-HT_6_ receptor knockout (5-HT_6_KO) mice have yielded varied results in ciliary morphology. One study reported that primary cilia in the cortex, hippocampus, and striatum of 5-HT_6_KO mice were significantly shorter compared to wild-type mice (Dupuy et al., 2023). In contrast, another reported no change in the ciliary length of primary cilia in the hippocampus of 5-HT_6_KO mice (Sun et al., 2021). Overexpression of homologous 5-HT_6_ receptors in primary cultured hippocampal neurons significantly increased cilia number and length, while knockdown only reduced ciliary length (Hu et al., 2017; Wang et al., 2019). High levels of heterologous 5-HT_6_ receptor expression increased the receptor’s presence outside of the primary cilia without altering the morphology of the primary cilia (Brodsky et al., 2017). In primary striatal neurons from 5-HT_6_KO mice, reintroduction of 5-HT_6_ receptors restored their primary ciliary localization without altering the overall ciliation rate, led to elongation of neuronal primary cilia, and significantly increased the average total dendritic length (Lesiak et al., 2018). Manipulating 5-HT_6_ receptor expression also affects axonal morphology. Overexpression of 5-HT_6_ receptors reduced axonal length to approximately 75% of that in wild-type neurons, whereas downregulation of 5-HT_6_ receptor expression led to increased axonal length (Wang et al., 2019). Loss of the 5-HT_6_ receptor results in significant functional alterations, including increased action potential firing frequency and a shortened axon initial segment in hippocampal pyramidal neurons, as well as heightened anxiety and cognitive impairments in 5-HT_6_KO mice (Sun et al., 2021). Similarly, an independent study reported that modulation of 5-HT_6_ receptor expression regulates axon initial segment length in cultured mouse hippocampal neurons, with overexpression increasing and knockout decreasing axon initial segment length (Wang et al., 2025). Conversely, overexpression of 5-HT_6_ receptors in mouse hippocampal neurons leads to decreased neuronal excitability, characterized by reduced peak amplitudes of single action potentials and fewer spikes compared to controls (Wang et al., 2019). Pharmacological studies have further explored the critical role of ciliary localization of the 5-HT_6_ receptor in its function. In cultured mouse striatal neurons, treatment with SB-399885 (5-HT_6_ receptor antagonist) shortened primary cilia with no impact on their ciliation. Crucially, this effect is absent in striatal neurons from 5-HT_6_KO, demonstrating that the drug’s effect is dependent on the presence of the receptor on the primary cilium. Treatment with WAY-208466 (5-HT_6_ receptor agonist) did not affect primary cilia length in cultured mouse striatal neurons (Brodsky et al., 2017). These studies collectively illustrate that the 5-HT_6_ receptor, acting from the primary cilium, serves as a regulator of neuronal structure, excitability, and function.

## Alterations of primary cilia in AD mouse models

**FIGURE 2:**
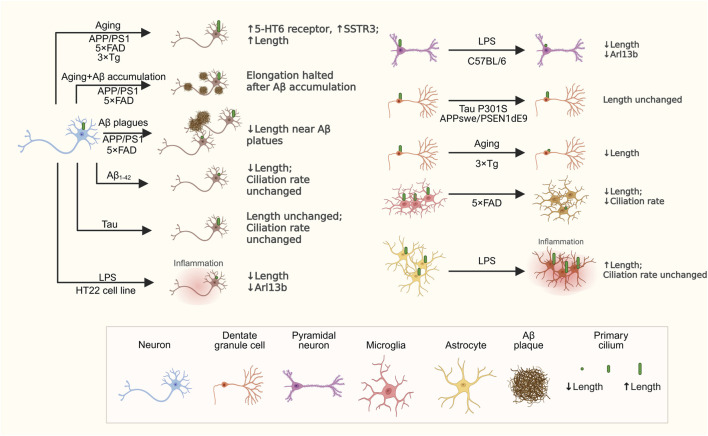
Changes in primary cilia across different cell types in AD models. This schematic highlights key changes in primary cilia among various cell types in AD models. Neurons experience age-related primary ciliary elongation, which is halted by Aβ accumulation. Treatment with Aβ_1-42_ reduces primary ciliary length, while tau overexpression does not influence primary cilia length or ciliation rate. Neuronal exposure to LPS also causes primary ciliary shortening. Microglia show decreased primary ciliary length and fewer primary cilia in 5xFAD. Astrocytes have longer primary cilia after LPS treatment, but their ciliation rate remains unchanged. These findings emphasize that primary cilia remodeling in AD is specific to cell type and context. Created in BioRender.

Although no studies have yet characterized primary cilia in postmortem human AD brain tissue, various transgenic mouse models have provided critical insights into primary ciliary alterations associated with AD pathology (Table 1; Figure 2).

**TABLE 1:** Alterations of primary cilia in AD models.

Model/Cell type	Age	Brain region	Cell type	Ciliary marker	Ciliary alterations^a^	Ref.
APP/PS1	6-month	Hippocampus	Neuron	AC3	• Length↑ • 5-HT_6_ receptor↑	Hu et al. (2017)
	12-month	Near Aβ plaques	Neuron	AC3	• Length↓compared to longer distances	Guo et al. (2025)
	12-month	Hippocampus	—	—	• Sstr3↑	
	2, 6, and 12-month	Hippocampal CA1	Neuron	AC3	• Length↑ with age, but elongation halts after Aβ accumulation onset	
APP^NL−G-F^	6, 13, and 15-month	Hippocampal CA1 or CA3	Neuron	AC3	• No age-dependent length change	Kobayashi et al. (2022)
APPswe/PSEN1dE9	6–8-month	Hippocampus	Dentate granule cell	Sstr3	• Length unchanged	Chakravarthy et al. (2012)
5×FAD	4-month	Near Aβ plaques	Neuron	AC3	• Length↓ compared to longer distances	Guo et al. (2025)
	1, 2, and 6-month	Hippocampal CA1	Neuron	AC3	• Length↑ with age, but elongation halts after Aβ accumulation onset	
	12-month	Hippocampus	—	—	• Sstr3↑	
	6-month	Cortex and lateral septum	Microglia	AC3	• Length↓ • Ciliation rate↓	Yeo et al. (2023)
Tau P301S	3–4-month	Hippocampus	Dentate granule cell	Sstr3	• Length unchanged	Chakravarthy et al. (2012)
3×Tg	12-month	Hippocampus	—	—	• Sstr3↑	Guo et al. (2025)
	6–24-month	Hippocampus	Dentate granule cell	Sstr3	• Length↓ with age	Chakravarthy et al. (2012)
Intracerebroventricular injection of LPS in C57BL/6 mice	8–10-week	Hippocampal CA1	Pyramidal Neuron	AC3	• Length↓ 1 day after injection • Arl13b ↓ 3 days after injection	Baek et al. (2017)
		Hippocampus	—	—	• Arl13b↓ 3 days after injection	
LPS-treated HT22	—	—	Mouse hippocampal neuronal cell line	Arl13b	• Length↓ after 1-h treatment • Arl13b↓ after 6-h treatment	Baek et al. (2017)
LPS-treated astrocyte	Postnatal-7 pup	—	Primary mouse astrocyte	Arl13b	• Length↑ in both astrocytes and C3-positive astrocytes • Ciliation rate unchanged	Muhamad et al. (2024)
Aβ_1-42_ treated neuron	Embryonic day 18 mouse embryos	—	Primary hippocampal neuron	AC3	• Length↓ • Ciliation rate unchanged	Guo et al. (2025)
Exogenous or endogenous overexpression of Tau in neuron					• Length unchanged • Ciliation rate unchanged	

^a^ Unless specified, these were compared to age-matched controls.

LPS, lipopolysaccharide; MIF, macrophage migration inhibitory factor.

Primary cilia length appears to be dynamically altered in response to Aβ plaque deposition, potentially reflecting a protective or compensatory mechanism against neurotoxic stress. In the brains of 12-month-old amyloid precursor protein/presenilin-1 (APP/PS1) and 4-month-old 5 × FAD mice, AC3-labeled neuronal primary cilia are significantly shorter in regions adjacent to Aβ plaques compared to more distant areas. In the hippocampal CA1 region of both models, neuronal primary cilia length gradually increases with age but ceases to elongate following the onset of Aβ accumulation (Guo et al., 2025).

Expression changes in primary cilia-localized GPCRs further highlight potential signaling dysregulation in AD mouse models, which may link ciliary alterations to broader pathological cascades. In APP/PS1 mice, hippocampal neurons exhibited elongated primary cilia with elevated expression of the ciliary 5-HT_6_ receptor. Notably, treatment with the 5-HT_6_ receptor antagonist SB-271046 ameliorated cognitive deficits in APP/PS1 mice (Hu et al., 2017), suggesting that dysregulated ciliary signaling may contribute to memory impairments. Similarly, Sstr3 expression was significantly elevated in the hippocampus of 12-month-old APP/PS1, 5 × FAD, and 3 × Tg mice relative to wild-type controls (Guo et al., 2025).

Aging effects on primary cilia in AD mouse models diverge from those in wild-type mice, indicating that AD pathology may override normal age-related ciliary dynamics, potentially accelerating neuronal vulnerability. For instance, APP^NL−G-F^ mice showed no age-dependent elongation of neuronal primary cilia in the CA1 or CA3 regions, unlike wild-type mice (Kobayashi et al., 2022). Similarly, Chakravarthy et al. reported that the length of Sstr3-labeled cilia in hippocampal granule cells remains unchanged in APPswe/PSEN1dE9 (6–8 months) and Tau P301S (3–4 months) mice compared to age-matched wild-type controls, whereas 3xTg mice exhibit significantly shorter cilia across multiple ages (6–24 months), pointing to model-specific disruptions (Chakravarthy et al., 2012).

Primary cilia alterations extend beyond neurons to immune cells, underscoring a potential role in AD-associated neuroinflammation that may integrate with other pathological features. In 6-month-old 5xFAD mice, AC3-positive microglial primary cilia were significantly reduced in the lateral septum and absent in the cortex, with microglia in the lateral septum exhibited shorter primary cilia compared to age-matched wild-type controls (Yeo et al., 2023). Further evidence from Baek et al. demonstrated that, 1 day after intracerebroventricular injection of lipopolysaccharide (LPS) in C57BL/6 mice, there was a reduction in both the length of AC3-labeled primary cilia and the expression of Arl13b in the hippocampus, accompanied by increased levels of inflammatory mediators, including Cox2 and iNOS. In contrast, toll-like receptor 4 (*Tlr4*) knockout mice show increased primary ciliary length and elevated Arl13b expression following LPS administration, without significant changes in Cox2 and iNOS levels. Similar effects occur *in vitro*: HT22 hippocampal cells exhibit shortened primary cilia after just 1 h of LPS exposure, with marked Arl13b reduction after 6 h, while LPS does not significantly alter Arl13b expression level in primary neurons from *Tlr4* ^*−/−*^ mice. In kinesin family member 3A (*Kif3a*) knockdown HT22 cells, 24-h LPS exposure fails to elicit an inflammatory response but results in a slight increase in Arl13b expression (Baek et al., 2017), suggesting that intact primary ciliary signaling may be required for efficient neuroinflammatory activation.

Emerging evidence also implicates primary cilia in protein clearance mechanisms, suggesting a speculative link to AD’s hallmark protein accumulations and cellular stress responses. In 5xFAD mice, macrophage migration inhibitory factor (MIF) localizes to both dystrophic axons near Aβ plaques and to the basal end of AC3-positive cilia in the hippocampus. The accumulation of MIF around the nucleus, together with ubiquitin, implies that primary cilia may facilitate neuronal clearance processes, potentially contributing to axonal dystrophy and extracellular vesicle accumulation within axonal spheroids (Jang et al., 2023).

Taken together, these studies suggest that primary cilia undergo context-dependent remodeling in AD models, which may serve as a marker of local pathological stress or a functional modulator of neuronal and glial responses. Whether these changes are protective, maladaptive, or a combination of both remains unresolved, but the accumulating evidence highlights primary cilia as active participants in AD pathogenesis, warranting further investigation into their mechanistic roles and therapeutic potential.

## The role of primary cilium in aging, cognition, and AD pathogenesis

Primary cilia have emerged as multifunctional organelles involved in proteostasis, neuroinflammation, and cognitive processes, thereby intersecting with multiple aspects of AD pathogenesis.

Aging is well established as a major risk factor for AD. Rivagorda et al. reported that the age-related decline in autophagy machinery in hippocampal neurons, which contributes to memory deficits, is associated with a marked reduction in primary cilium components, including Ift20, Ift88, and Kif3a, at both the mRNA and protein levels in the hippocampus of aged (16-month-old) C57 mice. Restoration of Ift20 expression in the hippocampus was sufficient to enhance autophagy activity in the CA3 region and reverse age-related cognitive deficits. Complementing these findings, the osteocalcin receptor GPR158 was found to be localized to the primary cilium of hippocampal neurons, where it mediates osteocalcin-regulated autophagy. Mechanistically, this effect depended on a primary cilium-CREB signaling pathway. Together, these findings emphasize the primary cilium–autophagy axis as a signaling route between blood-borne factors and neurons, providing new insight into the mechanisms underlying age-related cognitive decline (Rivagorda et al., 2025).

Disruption of primary cilia-related proteins across diverse brain cell types—including neurons, glia, and neural stem cells—results in cognitive deficits (Tables 2, 3). Hippocampal and cortical neuron-specific *Ift88* knockdown selectively impairs aversive and recognition memory while sparing spatial memory, and is accompanied by increased paired-pulse facilitation without changes in LTP, as shown by field excitatory postsynaptic potential recordings (Berbari et al., 2014). Beyond neurons, IFT88 is also critical in glial cells: astrocyte-specific *Ift88* knockout prevents the induction of C3-positive astrocytes by LPS or cytokine mixture (IL-1α, TNFα, and C1q) and protects against LPS-induced cognitive impairment observed in wild-type mice (Muhamad et al., 2024). Consistently, global *Ift88* knockout mice exhibit broad neurological impairments, including learning deficits, sleep architecture alteration, reduced electroencephalogram power, and diminished phase-amplitude coupling (Strobel et al., 2025). Other cilia-related proteins also impact memory. Knockout of AC3 impairs memory for temporally dissociative passive avoidance but does not affect contextual fear memory and short-term memory for novel object recognition (Wang et al., 2011). Conditional postnatal ablation of *Ift20* in mature dentate granule cells impairs hippocampus-dependent contextual memory and enhances LTP at mossy fiber synapses, a phenotype also observed with Kif3a disruption models (Rhee et al., 2016). Additionally, *Ift20* deletion in adult GFAP^+^ neural stem/progenitor cells reduces AC3-marked primary cilia in radial neural stem cells and decreases amplifying progenitor proliferation in hippocampal DG, without affecting quiescent progenitors or radial neural stem cells. This correlates with impaired spatial novelty recognition and heightened hippocampus-independent cue conditioning responses (Amador-Arjona et al., 2011). Collectively, these findings underscore the indispensable role of primary ciliary proteins in cognitive processes.

**TABLE 2:** Primary cilium-related genes and their functional roles in different cell.

Cell type	Primary cilium-related genes	Genetic manipulation	Experimental models	Functional outcomes	Ref.
Neuron	*Ift88*	knockdown	Primary mouse hippocampal neuron	• CD63-positive extracellular vesicle containing Aβ release↓	Jang et al. (2023)
Microglia			BV2	• Microglial activation markers↓ • Phagocytic activity↑ • Aβ-containing EV release↑	Yeo et al. (2023)
Astrocyte			Primary mouse astrocyte	• Cytokine mixture (IL-1α, TNFα, and C1q)- or LPS-induced C3-positive astrocytes↓	Muhamad et al. (2024)
Neuron	*Sstr3*	knockout	*Sstr3* ^−/−^ × 5 × FAD	• Apoptosis↑ in CA1 region • Number of swollen axonal structures surrounding Aβ plaques↑	Guo et al. (2025)
Microglia				• Activation↑ in CA1 region • Aggregation around Aβ↓	
Neural stem/progenitor cells	*Ift20*	knockout	*Ift20* ^*flox/flox*^ under control of the mGFAP-Cre	• AC3-marked primary cilia in radial neural stem cells↓ • Amplifying progenitor proliferation↓ in hippocampal DG • Quiescent progenitors or radial neural stem cells are not affected	Amador-Arjona et al. (2011)

**TABLE 3:** Primary cilium-related genes and their cognitive and pathological roles in mouse models.

Genes	Genetic models	Cognitive impacts	Roles in AD pathology	Ref.
*Sstr3*	*Sstr3* ^−/−^ × 5 × FAD	Not studied	• Aβ aggregation↑	Guo et al. (2025)
*Ift88*	Knockout mice	• Learning deficits • Electroencephalogram power↓ and diminished phase-amplitude coupling	—	Strobel et al. (2025)
	Hippocampal and cortical neuron-specific knockdown mice	• Aversive and recognition memory impaired • Paired-pulse facilitation ↑ with no changes in LTP	—	Berbari et al. (2014)
	Astrocyte-specific knockout mice	• LPS-induced cognitive impairment↓	—	Muhamad et al. (2024)
*AC3*	Knockout mice	• Memory for temporally dissociative passive avoidance↓ • Contextual fear memory and short-term memory for novel object recognition not affected	—	Wang et al. (2011)
*Kif3a*	Expression of a dominant negative Kif3a in mature dentate granule cells in mice, via stereotaxic injection of AAV9-DIO-dnKif3A into adult dentate gyrus	• Hippocampus-dependent contextual memory↓ • LTP at mossy fiber synapses↑	—	Rhee et al. (2016)
*Ift20*	Postnatal deletion: *IFT20* ^*foxl/flox*^ mice stereotaxically injected with AAV-CaMKII-eGFP-Cre virus at adult dentate gyrus	• Hippocampus-dependent contextual memory↓ • LTP at mossy fiber synapses↑	—	
	*Ift20* ^*flox/flox*^ under control of the mGFAP-Cre	• Spatial novelty recognition↓ • Hippocampus-independent cue conditioning responses↑	—	Amador-Arjona et al. (2011)
	Aged C57 mice (16-month-old)	• Autophagy machinery↓ in hippocampal neurons • Memory deficits • Restoring Ift20 levels in the hippocampus enhanced autophagy activity in the CA3 region and reverse age-related cognitive deficits	—	Rivagorda et al. (2025)

Primary cilia have been implicated in the regulation of extracellular vesicle (EV) transport, which is critical for the clearance of Aβ. Disruption of primary cilia by *Ift88* knockdown in primary hippocampal neurons reduced CD63-positive EV release, resulting in intracellular Aβ accumulation and enhanced extracellular Aβ uptake, thereby linking primary ciliary dysfunction to AD pathology (Jang et al., 2023). In microglia, *Ift88* knockdown reduced activation potential, enhanced phagocytic activity, and promoted Aβ-containing EV release, linking primary ciliary function to neuroinflammatory responses (Yeo et al., 2023). Together, these findings suggest that ciliary dysfunction may impair cooperative mechanisms of Aβ clearance across neuronal and glial compartments.

Ciliary morphology is directly influenced by amyloid pathology. Consistent with findings from AD model mice, *in vitro* data show that Aβ reduces primary cilia length. Exogenous Aβ_1_–_42_ exposure shortened the primary cilia of cultured mouse neurons without affecting the ciliation rates, whereas tau overexpression does not alters ciliary length or ciliation rate (Guo et al., 2025), suggesting a selective vulnerability to Aβ. Intriguingly, hippocampal neurons derived from multiple transgenic AD mouse models (APP/PS1, 5 × FAD, and 3 × Tg) exhibit elongated cilia during early *in vitro* development (Day 7), even before detectable Aβ deposition or altered secretion (Guo et al., 2025). This temporal dissociation suggests that primary ciliary remodeling may represent an early event preceding overt amyloid pathology.

Functional study suggests that primary ciliary GPCRs play a role in regulating plaque pathology. In offspring of *Sstr3* knockout mice crossed with 5 × FAD mice (Sstr3^−/−^ × 5 × FAD, 4 months old), the loss of the primary ciliary-localized receptor Sstr3 worsened Aβ accumulation, increased hippocampal neuronal apoptosis, and heightened microglial reactivity in the CA1 region. However, paradoxically, it reduced microglial clustering around Aβ plaques compared to 5 × FAD controls (Guo et al., 2025). This highlights that primary cilia-dependent GPCR signaling is involved in neuroimmune dynamics in AD.

Growing evidence indicates that primary cilia influence diverse pathological processes in AD—ranging from tau pathology and cerebrospinal fluid dysregulation to cellular responses to hypoxia—that collectively contribute to disease progression. Tau pathology, characterized by abnormal hyperphosphorylation and aggregation of tau protein, is a central hallmark of AD. The neurotrophin receptor p75^NTR^, which mediates Aβ-induced tau phosphorylation, may act in part through the primary ciliary compartment, suggesting a role for primary cilia in modulating tau-related signaling pathways (Chakravarthy et al., 2010; Shen et al., 2019). Disruption of CSF dynamics, which impairs the clearance of metabolic waste and Aβ, has also been implicated in AD. In the choroid plexus epithelium, primary cilia act as clustered chemosensors that regulate CSF production, and the neuropeptide FF receptor 2, localized to primary cilia, modulates fluid transcytosis in these cells (Narita et al., 2010). Cellular responses to hypoxia, another contributing factor in AD pathogenesis, are also linked to ciliary function. Under hypoxic conditions, neuronal primary cilia elongated and exhibited increased levels of Ift88 while hypoxia-inducible factor-2α accumulates within the primary cilium and interacts with Ift88 (Leu et al., 2023). In rat models of chronic cerebral hypoperfusion—a hypoxia-related condition—astrocytic primary cilia are shortened, but physical exercise restores ciliary length and density (Cao et al., 2022). Together, these findings suggest that primary cilia play diverse roles across multiple AD-related pathologies. However, direct studies in AD models remain limited, highlighting a significant knowledge gap and the need for further mechanistic investigation.

## Discussion and conclusion

Primary cilia are dynamic organelles that coordinate signaling, inter-organelle communication, and extracellular interactions across neurons, glia, and choroid plexus epithelial cells. They exhibit notable morphological diversity—including variations in length, spatial distribution, and microtubule architecture—which is further influenced by age, brain region, and cell type. Primary ciliary-localized GPCRs and other signaling proteins regulate key neural processes such as memory, synaptic plasticity, and neuronal excitability, yet the mechanistic consequences of their precise ciliary positioning remain incompletely understood.

In the context of AD, primary cilia are increasingly recognized as key players in disease-related processes. In transgenic AD models, neuronal and glial cilia exhibit region- and cell-type-specific morphological alterations, ranging from elongation to shortening, often associated with AD-linked genetic mutations (Table 1). Separately, disruption of ciliary components or signaling proteins—including Ift88, Ift20, Kif3a, and ciliary GPCRs—induces structural changes that are accompanied by impaired Aβ clearance, altered neuroinflammatory responses, and cognitive deficits (Tables 2, 3). Mechanistic evidence indicates that these effects involve ciliary GPCR signaling, autophagy-related pathways, and inter-organelle communication, particularly via extracellular vesicle trafficking, highlighting the central role of primary cilia in coordinating cellular functions relevant to AD pathology. These findings position primary cilia not merely as structural organelles but as active modulators of the interplay between Aβ accumulation, neuroinflammation, and neural circuit dysfunction. APP, which is cleaved by β- and γ-secretases to generate Aβ, has been shown to localize to primary cilia in NIH3T3 and HeLa cells (Vorobyeva and Saunders, 2018; Chebli et al., 2021). However, whether APP similarly localizes to primary cilia in brain-resident cells remains unclear. A deeper understanding of primary ciliary function across diverse AD models may reveal novel therapeutic targets for intervention.

Beyond Aβ pathology, primary cilia intersect with multiple AD-relevant processes, including tau signaling, cerebrospinal fluid regulation, autophagy, and cellular responses to hypoxia, reflecting their broad functional involvement. Primary cilia also engage in dynamic interactions with other cellular organelles, shaping cellular function under both physiological and pathological conditions. For example, astrocytic mitochondrial dysfunction disrupts ciliary structure (Ignatenko et al., 2023), and extracellular vesicles released from primary cilia carry biomarkers—such as NADPH-cytochrome P450 reductase, Topoisomerase II Alpha, and CD151—that interact with AD-related proteins including APP, Peptidyl-prolyl cis-trans isomerase NIMA-interacting 1, and Cathepsin B (Mohieldin et al., 2021). These observations suggest that primary cilia act not only as sensory hubs but also as mediators of inter-organelle crosstalk, potentially maintaining neuronal integrity and modulating neurodegenerative processes. Nevertheless, many findings remain correlational, and it is unclear whether ciliary remodeling represents a causal driver or a compensatory response to cellular stress. Discrepancies across models, differences between neuronal and glial cilia, and limited validation in human brain tissue further highlight the need for cautious interpretation. Collectively, these data indicate that primary cilia contribute to AD through diverse mechanisms, underscoring the need for further mechanistic studies to clarify their molecular roles and therapeutic potential.

Despite these gaps, primary cilia emerge as integrative hubs that coordinate intracellular signaling, inter-organelle communication, and extracellular interactions, thereby influencing cognitive function and modulating neurodegenerative processes. Future research should focus on: (1) dissecting cell-type- and brain region-specific ciliary mechanisms, including the role of individual ciliary proteins; (2) clarifying the temporal sequence of ciliary remodeling relative to amyloid and tau pathology and other AD-related stressors; and (3) evaluating the therapeutic potential of cilia-targeted interventions in translational and human-relevant models. Addressing these questions will be essential to establish whether primary cilia act as causal drivers, compensatory responders, or both in AD pathogenesis, and may ultimately reveal novel strategies for modulating neurodegeneration through ciliary pathways.

## References

[B1] Amador-Arjona A. Elliott J. Miller A. Ginbey A. Pazour G. J. Enikolopov G. (2011). Primary cilia regulate proliferation of amplifying progenitors in adult hippocampus: implications for learning and memory. J. Neurosci. 31 (27), 9933–9944. 10.1523/jneurosci.1062-11.2011 21734285 PMC3758574

[B2] Arellano J. I. Guadiana S. M. Breunig J. J. Rakic P. Sarkisian M. R. (2012). Development and distribution of neuronal cilia in mouse neocortex. J. Comp. Neurol. 520 (4), 848–873. 10.1002/cne.22793 22020803 PMC3325766

[B3] Arrojo E. D. R. Lev-Ram V. Tyagi S. Ramachandra R. Deerinck T. Bushong E. (2019). Age mosaicism across multiple scales in adult tissues. Cell Metab. 30 (2), 343–351.e343. 10.1016/j.cmet.2019.05.010 31178361 PMC7289515

[B4] Awata J. Takada S. Standley C. Lechtreck K. F. Bellvé K. D. Pazour G. J. (2014). NPHP4 controls ciliary trafficking of membrane proteins and large soluble proteins at the transition zone. J. Cell Sci. 127 (Pt 21), 4714–4727. 10.1242/jcs.155275 25150219 PMC4215714

[B5] Badgandi H. B. Hwang S.-h. Shimada I. S. Loriot E. Mukhopadhyay S. (2017). Tubby family proteins are adapters for ciliary trafficking of integral membrane proteins. J. Cell Biol. 216 (3), 743–760. 10.1083/jcb.201607095 28154160 PMC5350516

[B6] Baek H. Shin H. J. Kim J. J. Shin N. Kim S. Yi M. H. (2017). Primary cilia modulate TLR4-mediated inflammatory responses in hippocampal neurons. J. Neuroinflammation 14 (1), 189. 10.1186/s12974-017-0958-7 28927423 PMC5606072

[B7] Berbari N. F. Bishop G. A. Askwith C. C. Lewis J. S. Mykytyn K. (2007). Hippocampal neurons possess primary cilia in culture. J. Neurosci. Res. 85 (5), 1095–1100. 10.1002/jnr.21209 17304575

[B8] Berbari N. F. Lewis J. S. Bishop G. A. Askwith C. C. Mykytyn K. (2008). Bardet–Biedl syndrome proteins are required for the localization of G protein-coupled receptors to primary cilia. Proc. Natl. Acad. Sci. 105(11)**,** 4242–4246. 10.1073/pnas.0711027105 18334641 PMC2393805

[B9] Berbari N. F. Malarkey E. B. Yazdi S. M. McNair A. D. Kippe J. M. Croyle M. J. (2014). Hippocampal and cortical primary cilia are required for aversive memory in mice. PLoS One 9 (9), e106576. 10.1371/journal.pone.0106576 25184295 PMC4153651

[B10] Bishop G. A. Berbari N. F. Lewis J. Mykytyn K. (2007). Type III adenylyl cyclase localizes to primary cilia throughout the adult mouse brain. J. Comp. Neurol. 505 (5), 562–571. 10.1002/cne.21510 17924533

[B11] Brailov I. Bancila M. Brisorgueil M. J. Miquel M. C. Hamon M. Vergé D. (2000). Localization of 5-HT(6) receptors at the plasma membrane of neuronal cilia in the rat brain. Brain Res. 872 (1-2), 271–275. 10.1016/s0006-8993(00)02519-1 10924708

[B12] Brodsky M. Lesiak A. J. Croicu A. Cohenca N. Sullivan J. M. Neumaier J. F. (2017). 5-HT(6) receptor blockade regulates primary cilia morphology in striatal neurons. Brain Res. 1660, 10–19. 10.1016/j.brainres.2017.01.010 28087224 PMC5392252

[B13] Cao W. Lin J. Xiang W. Liu J. Wang B. Liao W. (2022). Physical exercise-induced astrocytic neuroprotection and cognitive improvement through primary cilia and mitogen-activated protein kinases pathway in rats with chronic cerebral hypoperfusion. Front. Aging Neurosci. 14, 866336. 10.3389/fnagi.2022.866336 35721009 PMC9198634

[B14] Chakravarthy B. Gaudet C. Ménard M. Atkinson T. Chiarini A. Dal Prà I. (2010). The p75 neurotrophin receptor is localized to primary cilia in adult murine hippocampal dentate gyrus granule cells. Biochem. Biophysical Res. Commun. 401 (3), 458–462. 10.1016/j.bbrc.2010.09.081 20875398

[B15] Chakravarthy B. Gaudet C. Ménard M. Brown L. Atkinson T. LaFerla F. M. (2012). Reduction of the immunostainable length of the hippocampal dentate granule cells’ primary cilia in 3xAD-transgenic mice producing human Aβ1-42 and tau. Biochem. Biophysical Res. Commun. 427 (1), 218–222. 10.1016/j.bbrc.2012.09.056 22995307

[B16] Chebli J. Rahmati M. Lashley T. Edeman B. Oldfors A. Zetterberg H. (2021). The localization of amyloid precursor protein to ependymal cilia in vertebrates and its role in ciliogenesis and brain development in zebrafish. Sci. Rep. 11 (1), 19115. 10.1038/s41598-021-98487-7 34580355 PMC8476544

[B17] Diniz G. B. Battagello D. S. Klein M. O. Bono B. S. M. Ferreira J. G. P. Motta-Teixeira L. C. (2020). Ciliary melanin-concentrating hormone receptor 1 (MCHR1) is widely distributed in the murine CNS in a sex-independent manner. J. Neurosci. Res. 98 (10), 2045–2071. 10.1002/jnr.24651 32530066

[B18] Domire J. S. Mykytyn K. (2009). Markers for neuronal cilia. Methods Cell Biol. 91, 111–121. 10.1016/s0091-679x(08)91006-2 20409783

[B19] Dupuy V. Prieur M. Pizzoccaro A. Margarido C. Valjent E. Bockaert J. (2023). Spatiotemporal dynamics of 5-HT(6) receptor ciliary localization during mouse brain development. Neurobiol. Dis. 176, 105949. 10.1016/j.nbd.2022.105949 36496200

[B20] Einstein E. B. Patterson C. A. Hon B. J. Regan K. A. Reddi J. Melnikoff D. E. (2010). Somatostatin signaling in neuronal cilia is critical for object recognition memory. J. Neurosci. 30 (12), 4306–4314. 10.1523/jneurosci.5295-09.2010 20335466 PMC3842454

[B21] Green J. A. Gu C. Mykytyn K. (2012). Heteromerization of ciliary G protein-coupled receptors in the mouse brain. PLoS One 7 (9), e46304. 10.1371/journal.pone.0046304 23029470 PMC3459911

[B22] Guadiana S. M. Parker A. K. Filho G. F. Sequeira A. Semple-Rowland S. Shaw G. (2016). Type 3 adenylyl cyclase and somatostatin receptor 3 expression persists in aged rat neocortical and hippocampal neuronal cilia. Front. Aging Neurosci. 8, 127. 10.3389/fnagi.2016.00127 27303293 PMC4885836

[B23] Guo A. Wang H. Zhang Y. Huang H. (2025). Changes of the primary cilia in Alzheimer's disease pathogenesis. Eur. J. Neurosci. 61 (9), e70125. 10.1111/ejn.70125 40329506

[B24] Hamon M. Doucet E. Lefèvre K. Miquel M. C. Lanfumey L. Insausti R. (1999). Antibodies and antisense oligonucleotide for probing the distribution and putative functions of central 5-HT6 receptors. Neuropsychopharmacology 21 (2 Suppl. l), 68s–76s. 10.1016/s0893-133x(99)00044-5 10432491

[B25] Händel M. Schulz S. Stanarius A. Schreff M. Erdtmann-Vourliotis M. Schmidt H. (1999). Selective targeting of somatostatin receptor 3 to neuronal cilia. Neuroscience 89 (3), 909–926. 10.1016/S0306-4522(98)00354-6 10199624

[B26] Hasenpusch-Theil K. Theil T. (2021). The multifaceted roles of primary cilia in the development of the cerebral cortex. Front. Cell Dev. Biol. 9, 630161–632021. 10.3389/fcell.2021.630161 33604340 PMC7884624

[B27] Hesketh S. J. Mukhopadhyay A. G. Nakamura D. Toropova K. Roberts A. J. (2022). IFT-A structure reveals carriages for membrane protein transport into cilia. Cell 185 (26), 4971–4985.e16. 10.1016/j.cell.2022.11.010 36462505

[B28] Hsiao Y. C. Muñoz-Estrada J. Tuz K. Ferland R. J. (2021). The transition zone protein AHI1 regulates neuronal ciliary trafficking of MCHR1 and its downstream signaling pathway. J. Neurosci. 41 (17), 3932–3943. 10.1523/jneurosci.2993-20.2021 33741721 PMC8084322

[B29] Hu L. Wang B. Zhang Y. (2017). Serotonin 5-HT6 receptors affect cognition in a mouse model of Alzheimer's disease by regulating cilia function. Alzheimers Res. Ther. 9 (1), 76. 10.1186/s13195-017-0304-4 28931427 PMC5607612

[B30] Ignatenko O. Malinen S. Rybas S. Vihinen H. Nikkanen J. Kononov A. (2023). Mitochondrial dysfunction compromises ciliary homeostasis in astrocytes. J. Cell Biol. 222 (1), e202203019. 10.1083/jcb.202203019 36383135 PMC9674092

[B31] Jang J. Yeo S. Baek S. Jung H. J. Lee M. S. Choi S. H. (2023). Abnormal accumulation of extracellular vesicles in hippocampal dystrophic axons and regulation by the primary cilia in Alzheimer’s disease. Acta Neuropathol. Commun. 11 (1), 142. 10.1186/s40478-023-01637-3 37667395 PMC10478284

[B32] Kasahara K. Miyoshi K. Murakami S. Miyazaki I. Asanuma M. (2014). Visualization of astrocytic primary cilia in the mouse brain by immunofluorescent analysis using the cilia marker Arl13b. Acta Med. Okayama 68 (6), 317–322. 10.18926/amo/53020 25519025

[B33] Klena N. Pigino G. (2022). Structural biology of cilia and intraflagellar transport. Annu. Rev. Cell Dev. Biol. 38, 103–123. 10.1146/annurev-cellbio-120219-034238 35767872

[B34] Knopman D. S. Amieva H. Petersen R. C. Chételat G. Holtzman D. M. Hyman B. T. (2021). Alzheimer disease. Nat. Rev. Dis. Prim. 7 (1), 33. 10.1038/s41572-021-00269-y 33986301 PMC8574196

[B35] Kobayashi Y. Okada T. Miki D. Sekino Y. Koganezawa N. Shirao T. (2021). Properties of primary cilia in melanin-concentrating hormone receptor 1-bearing hippocampal neurons *in vivo* and *in vitro* . Neurochem. Int. 142, 104902. 10.1016/j.neuint.2020.104902 33197527

[B36] Kobayashi Y. Kohbuchi S. Koganezawa N. Sekino Y. Shirao T. Saido T. C. (2022). Impairment of ciliary dynamics in an APP knock-in mouse model of Alzheimer's disease. Biochem. Biophysical Res. Commun. 610, 85–91. 10.1016/j.bbrc.2022.04.050 35453040

[B37] Kumamoto N. Gu Y. Wang J. Janoschka S. Takemaru K. Levine J. (2012). A role for primary cilia in glutamatergic synaptic integration of adult-born neurons. Nat. Neurosci. 15 (3), 399–405. 10.1038/nn.3042 22306608 PMC3288565

[B38] Lechtreck K. F. (2015). IFT-cargo interactions and protein transport in cilia. Trends Biochem. Sci. 40 (12), 765–778. 10.1016/j.tibs.2015.09.003 26498262 PMC4661101

[B39] Lesiak A. J. Brodsky M. Cohenca N. Croicu A. G. Neumaier J. F. (2018). Restoration of physiological expression of 5-HT(6) receptor into the primary cilia of null mutant neurons lengthens both primary cilia and dendrites. Mol. Pharmacol. 94 (1), 731–742. 10.1124/mol.117.111583 29678909 PMC5987994

[B40] Leu T. Denda J. Wrobeln A. Fandrey J. (2023). Hypoxia-inducible factor-2alpha affects the MEK/ERK signaling pathway via primary cilia in connection with the intraflagellar transport protein 88 homolog. Mol. Cell Biol. 43 (4), 174–183. 10.1080/10985549.2023.2198931 37074220 PMC10153011

[B41] Liao H. Huang J. Liu J. Chen Y. Zhu H. Li X. (2023). Resveratrol inhibits activation of microglia after stroke through triggering translocation of Smo to primary cilia. J. Pers. Med. 13 (2), 268. 10.3390/jpm13020268 36836502 PMC9961736

[B42] Ma R. Kutchy N. A. Chen L. Meigs D. D. Hu G. (2022). Primary cilia and ciliary signaling pathways in aging and age-related brain disorders. Neurobiol. Dis. 163, 105607. 10.1016/j.nbd.2021.105607 34979259 PMC9280856

[B43] Mohieldin A. M. Alachkar A. Yates J. Nauli S. M. (2021). Novel biomarkers of ciliary extracellular vesicles interact with ciliopathy and Alzheimer's associated proteins. Commun. Integr. Biol. 14 (1), 264–269. 10.1080/19420889.2021.2017099 34992713 PMC8726672

[B44] Muhamad N. A. Masutani K. Furukawa S. Yuri S. Toriyama M. Matsumoto C. (2024). Astrocyte-specific inhibition of the primary cilium suppresses C3 expression in reactive astrocyte. Cell Mol. Neurobiol. 44 (1), 48. 10.1007/s10571-024-01482-5 38822888 PMC11144130

[B45] Mukhopadhyay S. Wen X. Chih B. Nelson C. D. Lane W. S. Scales S. J. (2010). TULP3 bridges the IFT-A complex and membrane phosphoinositides to promote trafficking of G protein-coupled receptors into primary cilia. Genes Dev. 24 (19), 2180–2193. 10.1101/gad.1966210 20889716 PMC2947770

[B46] Narita K. Kawate T. Kakinuma N. Takeda S. (2010). Multiple primary cilia modulate the fluid transcytosis in choroid plexus epithelium. Traffic 11 (2), 287–301. 10.1111/j.1600-0854.2009.01016.x 19958467

[B47] Nixon R. A. (2017). Amyloid precursor protein and endosomal-lysosomal dysfunction in Alzheimer's disease: inseparable partners in a multifactorial disease. FASEB J. 31 (7), 2729–2743. 10.1096/fj.201700359 28663518 PMC6137496

[B48] Pala R. Alomari N. Nauli S. M. (2017). Primary cilium-dependent signaling mechanisms. Int. J. Mol. Sci. 18 (11), 2272. 10.3390/ijms18112272 29143784 PMC5713242

[B49] Park K. Leroux M. R. (2022). Composition, organization and mechanisms of the transition zone, a gate for the cilium. EMBO Rep. 23 (12), e55420. 10.15252/embr.202255420 36408840 PMC9724682

[B50] Plotnikova O. V. Pugacheva E. N. Golemis E. A. (2009). “Chapter 7 - primary cilia and the cell cycle,” in Methods in cell biology. Editor Sloboda R. D. (Academic Press), 137–160. 10.1016/S0091-679X(08)94007-3 PMC2852269 20362089

[B51] Reiter J. F. Leroux M. R. (2017). Genes and molecular pathways underpinning ciliopathies. Nat. Rev. Mol. Cell Biol. 18 (9), 533–547. 10.1038/nrm.2017.60 28698599 PMC5851292

[B52] Rhee S. Kirschen G. W. Gu Y. Ge S. (2016). Depletion of primary cilia from mature dentate granule cells impairs hippocampus-dependent contextual memory. Sci. Rep. 6, 34370. 10.1038/srep34370 27678193 PMC5039642

[B53] Rivagorda M. Romeo-Guitart D. Blanchet V. Mailliet F. Boitez V. Barry N. (2025). A primary cilia-autophagy axis in hippocampal neurons is essential to maintain cognitive resilience. Nat. Aging 5 (3), 450–467. 10.1038/s43587-024-00791-0 39984747 PMC11922775

[B54] Schulz S. Händel M. Schreff M. Schmidt H. Höllt V. (2000). Localization of five somatostatin receptors in the rat central nervous system using subtype-specific antibodies. J. Physiology-Paris 94 (3), 259–264. 10.1016/S0928-4257(00)00212-6 11088003

[B55] Shen L.-L. Li W.-W. Xu Y.-L. Gao S.-H. Xu M.-Y. Bu X.-L. (2019). Neurotrophin receptor p75 mediates amyloid β-induced tau pathology. Neurobiol. Dis. 132, 104567. 10.1016/j.nbd.2019.104567 31394202

[B56] Sheu S. H. Upadhyayula S. Dupuy V. Pang S. Deng F. Wan J. (2022). A serotonergic axon-cilium synapse drives nuclear signaling to alter chromatin accessibility. Cell 185 (18), 3390–3407.e18. 10.1016/j.cell.2022.07.026 36055200 PMC9789380

[B57] Sipos É. Komoly S. Ács P. (2018). Quantitative comparison of primary cilia marker expression and length in the mouse brain. J. Mol. Neurosci. 64 (3), 397–409. 10.1007/s12031-018-1036-z 29464516

[B58] Strobel M. R. Zhou Y. Qiu L. Hofer A. M. Chen X. (2025). Temporal ablation of the ciliary protein IFT88 alters normal brainwave patterns. Sci. Rep. 15 (1), 347. 10.1038/s41598-024-83432-1 39747370 PMC11697071

[B59] Sun X. Haley J. Bulgakov O. V. Cai X. McGinnis J. Li T. (2012). Tubby is required for trafficking G protein-coupled receptors to neuronal cilia. Cilia 1 (1), 21. 10.1186/2046-2530-1-21 23351594 PMC3599646

[B60] Sun S. Fisher R. L. Bowser S. S. Pentecost B. T. Sui H. (2019). Three-dimensional architecture of epithelial primary cilia. Proc. Natl. Acad. Sci. U. S. A. 116 (19), 9370–9379. 10.1073/pnas.1821064116 31004057 PMC6511023

[B61] Sun Z. Wang B. Chen C. Li C. Zhang Y. (2021). 5-HT6R null mutatrion induces synaptic and cognitive defects. Aging Cell 20 (6), e13369. 10.1111/acel.13369 33960602 PMC8208783

[B62] Tereshko L. Gao Y. Cary B. A. Turrigiano G. G. Sengupta P. (2021). Ciliary neuropeptidergic signaling dynamically regulates excitatory synapses in postnatal neocortical pyramidal neurons. eLife 10, e65427. 10.7554/eLife.65427 33650969 PMC7952091

[B63] van den Hoek H. Klena N. Jordan M. A. Alvarez Viar G. Righetto R. D. Schaffer M. (2022). *In situ* architecture of the ciliary base reveals the stepwise assembly of intraflagellar transport trains. Science 377 (6605), 543–548. 10.1126/science.abm6704 35901159

[B64] Vien T. N. Ta M. C. Kimura L. F. Onay T. DeCaen P. G. (2023). Primary cilia TRP channel regulates hippocampal excitability. Proc. Natl. Acad. Sci. U. S. A. 120 (22), e2219686120. 10.1073/pnas.2219686120 37216541 PMC10235993

[B65] Vorobyeva A. G. Saunders A. J. (2018). Amyloid-β interrupts canonical Sonic hedgehog signaling by distorting primary cilia structure. Cilia 7 (1), 5. 10.1186/s13630-018-0059-y 30140428 PMC6098584

[B66] Wang Z. Phan T. Storm D. R. (2011). The type 3 adenylyl cyclase is required for novel object learning and extinction of contextual memory: role of cAMP signaling in primary cilia. J. Neurosci. 31 (15), 5557–5561. 10.1523/jneurosci.6561-10.2011 21490195 PMC3091825

[B67] Wang B. Hu L. Sun Z. Zhang Y. (2019). Cilia function is associated with axon initial segment morphology. Biochem. Biophysical Res. Commun. 516 (1), 15–21. 10.1016/j.bbrc.2019.05.172 31186137

[B68] Wang L. Guo Q. Acharya S. Zheng X. Huynh V. Whitmore B. (2024). Primary cilia signaling in astrocytes mediates development and regional-specific functional specification. Nat. Neurosci. 27 (9), 1708–1720. 10.1038/s41593-024-01726-z 39103557

[B69] Wang H. Li Y. Li X. Sun Z. Yu F. Pashang A. (2025). The primary cilia are associated with the axon initial segment in neurons. Adv. Sci. (Weinh) 12 (9), e2407405. 10.1002/advs.202407405 39804991 PMC11884599

[B70] Wei Q. Zhang Y. Li Y. Zhang Q. Ling K. Hu J. (2012). The BBSome controls IFT assembly and turnaround in cilia. Nat. Cell Biol. 14 (9), 950–957. 10.1038/ncb2560 22922713 PMC3434251

[B71] Wheatley D. N. Wang A. M. Strugnell G. E. (1996). Expression of primary cilia in mammalian cells. Cell Biol. Int. 20 (1), 73–81. 10.1006/cbir.1996.0011 8936410

[B72] Wingfield J. L. Lechtreck K. F. Lorentzen E. (2018). Trafficking of ciliary membrane proteins by the intraflagellar transport/BBSome machinery. Essays Biochem. 62 (6), 753–763. 10.1042/ebc20180030 30287585 PMC6737936

[B73] Wu J. Y. Cho S.-J. Descant K. Li P. H. Shapson-Coe A. Januszewski M. (2024). Mapping of neuronal and glial primary cilia contactome and connectome in the human cerebral cortex. Neuron 112 (1), 41–55.e3. 10.1016/j.neuron.2023.09.032 37898123 PMC10841524

[B74] Ye F. Nager A. R. Nachury M. V. (2018). BBSome trains remove activated GPCRs from cilia by enabling passage through the transition zone. J. Cell Biol. 217 (5), 1847–1868. 10.1083/jcb.201709041 29483145 PMC5940304

[B75] Yeo S. Jang J. Jung H. J. Lee H. Choe Y. (2023). Primary cilia-mediated regulation of microglial secretion in Alzheimer’s disease. Front. Mol. Biosci. 10, 1250335. 10.3389/fmolb.2023.1250335 37942288 PMC10627801

[B76] Yoshimura K. Kawate T. Takeda S. (2011). Signaling through the primary cilium affects glial cell survival under a stressed environment. Glia 59 (2), 333–344. 10.1002/glia.21105 21125655

[B77] Zaidi D. Chinnappa K. Francis F. (2022). Primary cilia influence progenitor function during cortical development. Cells 11 (18), 2895. 10.3390/cells11182895 36139475 PMC9496791

